# A Meta-Analysis of the Val158Met COMT Polymorphism and Violent Behavior in Schizophrenia

**DOI:** 10.1371/journal.pone.0043423

**Published:** 2012-08-14

**Authors:** Jay P. Singh, Jan Volavka, Pál Czobor, Richard A. Van Dorn

**Affiliations:** 1 Department of Mental Health Law and Policy, University of South Florida, Tampa, Florida, United States of America; 2 Department of Psychiatry, New York University School of Medicine, New York, New York, United States of America; 3 Nathan Kline Institute for Psychiatric Research, Orangeburg, New York, United States of America; 4 Department of Psychiatry and Psychotherapy, Semmelweis University, Budapest, Hungary; 5 Research Triangle Institute International, Durham, North Carolina, United States of America; Baylor College of Medicine, United States of America

## Abstract

We conducted a meta-analysis of studies examining the association between the Val158Met COMT polymorphism and violence against others in schizophrenia. A systematic search current to November 1, 2011 was conducted using MEDLINE, EMBASE, CINAHL, PsycINFO, ProQuest, and the National Criminal Justice Reference Service and identified 15 studies comprising 2,370 individuals with schizophrenia for inclusion. Bivariate analyses of study sensitivities and specificities were conducted. This methodology allowed for the calculation of pooled diagnostic odds ratios (DOR). Evidence of a significant association between the presence of a Met allele and violence was found such that men's violence risk increased by approximately 50% for those with at least one Met allele compared with homozygous Val individuals (DOR = 1.45; 95% CI = 1.05–2.00; *z* = 2.37, *p* = 0.02). No significant association between the presence of a Met allele and violence was found for women or when outcome was restricted to homicide. We conclude that male schizophrenia patients who carry the low activity Met allele in the COMT gene are at a modestly elevated risk of violence. This finding has potential implications for the pharmacogenetics of violent behavior in schizophrenia.

## Introduction

Schizophrenia elevates the risk of violent behavior [1]. Violence risk is a frequent reason for hospital admission, delays hospital discharge, complicates patient care, increases caregiver burden, leads to arrest and incarceration, and thus increases the stigma as well as the financial cost of the disease. For these reasons, violence in schizophrenia constitutes a major public health concern [2].

Violence is a complicated phenomenon that results from the interaction between many biological and social factors. Although serotonin is the principal neurotransmitter in the regulation of violence, dopamine and noradrenaline are also involved [3]. Enhancement of central dopaminergic or noradrenergic function facilitates aggressive behavior in most (but not all) animal studies [4]. Drugs that increase central dopaminergic transmission, such as amphetamines and cocaine, may elicit psychosis with violent behavior [2]. Furthermore, drugs that diminish noradrenergic activity (such as propranolol) have antiaggressive effects in humans [5], [6]. Thus, the preponderance of the evidence suggests that catecholamines generally enhance violence.

Catechol-*O*-methyltransferase (COMT) is one of the enzymes responsible for the catabolism of dopamine and noradrenaline in the brain. A common biallelic single nucleotide polymorphism, involving a Val (valine) to Met (methionine) substitution at codon 158 of the COMT gene (rs4680) has been identified and localized to chromosome 22q11.1-q11.2 [7]. The Val allele at this locus is associated with high enzymatic activity, whereas the Met allele is associated with low enzymatic activity. Homozygosity for the Met allele yields a 3- to 4-fold reduction in COMT activity relative to Val homozygotes, with heterozygotes demonstrating intermediate activity.

Male heterozygous COMT knockout mice have been shown to exhibit increased aggressive behavior [8]. When eight mouse strains were ranked according to their aggressivity, the ranking correlated with the expression of the COMT gene in the hippocampus: the lower the level of expression, the more aggressive the strain [9]. (Expression was assessed by quantifying the mRNA, genotypes were not reported [9]).

Thus, consistent with the enhancing effects of catecholamines on aggression, low expression of the COMT is associated with increased aggression in animal models. Based on the findings discussed above, it would seem appropriate to hypothesize that, in general, the COMT polymorphism would exert an effect in humans such that the Met allele would be associated with increased violent behavior. This would also be expected in schizophrenia patients. However, the original reason for COMT genotyping in these patients was to study association between this genotype and the diagnosis of schizophrenia rather than violence. Serendipitously, it was noted that the Met allele was associated with dangerousness [10], rather than with the diagnosis of schizophrenia [11].

Subsequent studies of violence and the COMT polymorphism in schizophrenia were conducted to replicate and expand these findings. The studies were heterogeneous in diagnoses and outcome variables. The samples included patients with schizophrenia, schizoaffective disorder, delusional disorder, and other diagnoses. The outcome variables combined physical violence against others with self-harm, violence against objects, verbal aggression, and threats. These differences make the findings of available studies difficult to compare directly, and may be responsible for their conflicting results. We present a meta-analysis of these studies focused on one diagnosis (schizophrenia) and one outcome (physical violence against others).

## Methods

### Study protocol

The Preferred Reporting Items for Systematic Reviews and Meta-analyses (PRISMA) Statement [12], a 27-item checklist of review characteristics, was followed to enable a transparent and consistent reporting of results.

**Figure 1:**
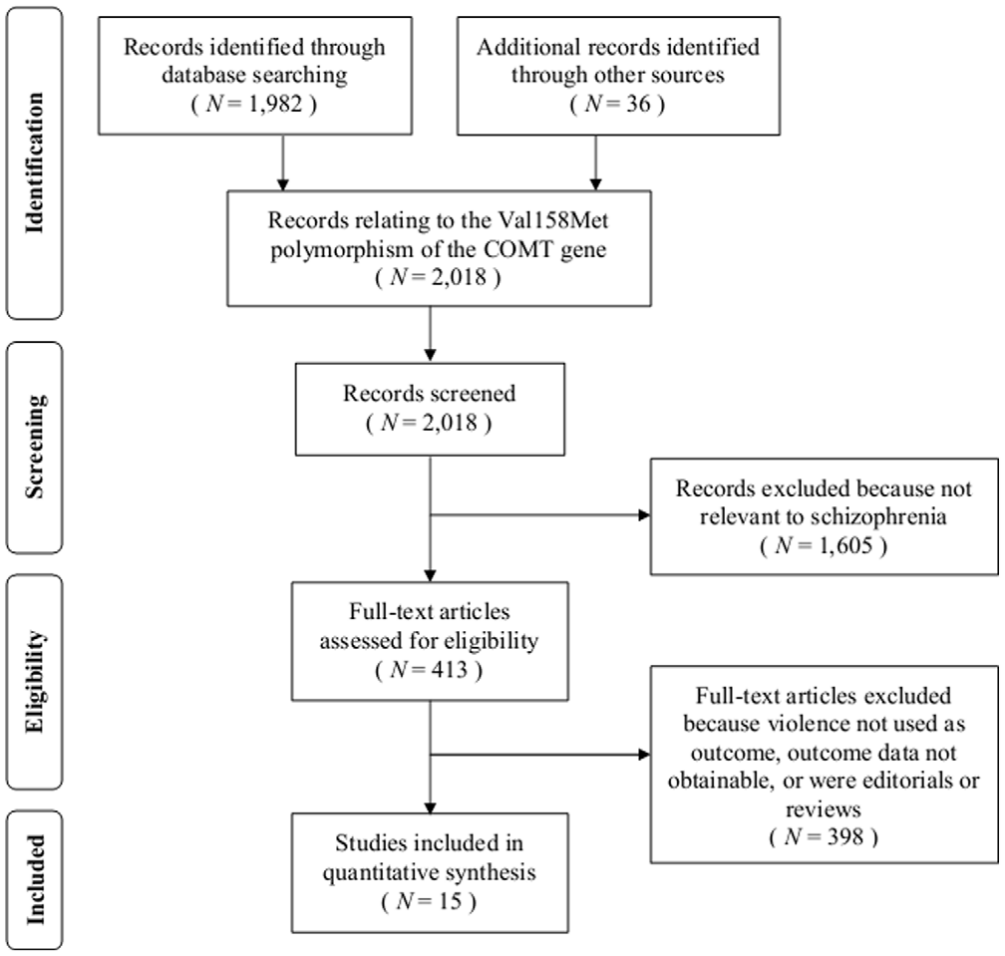
Systematic search for studies investigating the association between the Val158Met COMT polymorphism and violence.

**Table 1:** Table **1.** Descriptive characteristics of studies investigating the association between the Met allele of the Val158Met polymorphism and violence.

Study	Country	Dx	*N*	% Male	% Alc	% Drug	Mean age in years (SD)
Gu et al [55]	China	DSM-IV	584	100	0	0	34.9 (–)
Han et al [23]	S Korea	DSM-IV	29	100	0	0	26.4 (4.4)
Han et al [24]	S Korea	DSM-IV	168	100	0	0	38.9 (10.7)
Hong et al [52]	S Korea	DSM-IV	193	100	–	–	39.3 (8.6)
Jones et al [56]	UK	DSM-IV	180	75.6	–	–	–
Kim et al [32]	S Korea	DSM-IV	165	58.2	–	–	38.4 (10.1)
Koh et al [57]	S Korea	DSM-IV	99	85.9	0	0	36.8 (6.9)
Kotler et al [58]	Israel	ICD-10	92	–	–	–	44.6 (–)
Lachman et al [7]	USA	DSM-IV	31	71.0	80.6	80.6	42.6 (8.9)
Liou et al [59]	Taiwan	DSM-IV	198	47.5	0	0	37.8 (12.1)
Park et al [60]	S Korea	DSM-IV	103	61.2	–	–	42.2 (7.8)
Strous et al [61]	Israel	DSM-IV	122	77.0	–	–	–
Tosato et al [33]	Italy	ICD-10	80	53.8	3.8	1.3	42.1 (12.2)
Xiong et al [31]	China	CCMD-3	179	100	0	0	33.2 (–)
Zammit et al [62]	UK	DSM-IV	147	70.7	30.6	23.8	45.4 (13.8)

**Note:** –  =  data not obtainable; UK  =  United Kingdom; S Korea  =  South Korea; Dx  =  diagnostic system; DSM-IV  =  Diagnostic and Statistical Manual of Mental Disorders (4th edition); ICD-10 =  International Classification of Diseases (10th edition); CCMD-3 =  Chinese Classification of Mental Disorders; n  =  sample size; % Male  =  percentage of male participants; % Alc  =  percentage of participants with lifetime diagnoses of alcohol abuse or dependence; % Drug  =  percentage of participants with lifetime diagnoses of drug (non-alcohol) abuse or dependence. Total sample sizes and the number of participants within each genotype are not always equivalent to those that appear in published articles, as only individuals diagnosed with schizophrenia were included in the present review and outcome data could not always be obtained for all participants.

**Table 2:** Outcome characteristics of studies investigating the association between the Met allele of the Val158Met polymorphism and violence.

Study	*n*_Val/Val_ Viol	*n*_Val/Val_ Not Viol	*n*_Val/Met_ Viol	*n*_Val/Met_ Not Viol	*n*_Met/Met_ Viol	*n*_Met/Met_ Not Viol	Mean TARin months (SD)	Setting of outcome	Outcome
Gu et al [55]	128	156	104	148	20	28	1.0 (0)	Community	Homicide or malicious injury
Han et al [23]	5	14	4	4	1	1	100.8 (62.4)	Mixed	OAS4 aggression
Han et al [24]	17	74	16	48	6	7	13.6 (7.6)	Community	OAS4 aggression
Hong et al [52]	56	72	32	24	5	4	117.6 (90)	Institution	Homicide
Jones et al [56]	22	21	28	63	18	28	–	Mixed	OAS4 aggression
Kim et al [32]	35	66	25	27	1	11	0.5 (0)	Mixed	At least two serious assaults
Koh et al [57]	32	24	20	13	5	5	441.6 (82.8)	Community	Homicide
Kotler et al [58]	6	16	10	30	14	16	–	Community	Homicide
Lachman et al [7]	2	7	9	4	6	3	511.2 (106.8)	Mixed	At least two assaults
Liou et al [59]	46	72	22	46	4	8	0.47 (0)	Community	Physical aggression
Park et al [60]	26	30	17	21	4	5	–	Mixed	At least two violent episodes
Strous et al [61]	7	34	19	33	19	10	–	Mixed	Physical fight or assault
Tosato et al [33]	4	21	3	35	5	12	72.0 (0)	Mixed	OAS4 aggression
Xiong et al [31]	59	50	35	25	8	2	0.5 (0)	Community	MOAS4 aggression
Zammit et al [62]	12	28	23	44	11	29	244.8 (154.8)	Mixed	OAS4 aggression

**Note**: –  =  data not obtainable; n  =  sample size; Viol  =  violent; TAR  =  time at risk; SD  =  standard deviation; Mixed  =  both community and intra-institutional violence used as outcome; OAS4 =  physical aggression checklist of the Overt Aggression Scale [63]; MOAS4 =  physical aggression checklist of the Modified Overt Aggression Scale [64]; % Viol  =  base rate of violence. Total sample sizes and the number of participants with each genotype are not always equivalent to those that appear in published articles, as only individuals diagnosed with schizophrenia were included in the present review and outcome data could not always be obtained for all participants.

**Figure 2:**
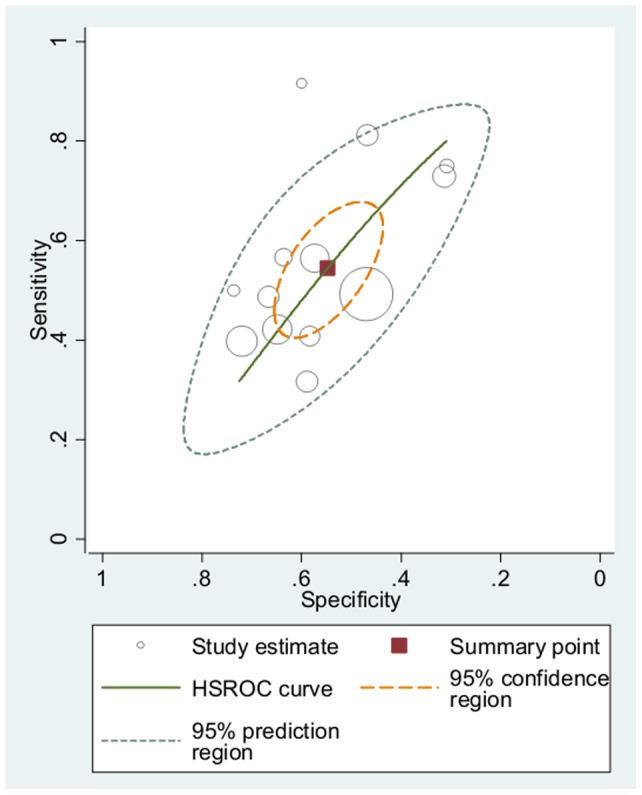
Hierarchical summary ROC curve predicting violence in men with at least one Met allele.

**Table 3:** Investigating the association between the Met allele of the Val158Met polymorphism and violence.

		Physical violence against others			Homicide only		
Genotypic comparison	Effect size	All	Men	Women	All	Men	Women
Val/Met+Met/Met vs. Val/Val	DOR (95% CI)	1.20 (0.91–1.59)	**1.45** * **(1.05**–**2.00)**	0.96 (0.51–1.79)	0.99 (0.76–1.29)	0.96 (0.71–1.30)	0.84 (0.31–2.26)
	*I^2^* (95% CI)	49.70 (8.82–72.24)	44.60 (0.01–71.01)	6.30 (0.01–42.62)	0.01 (0.01–41.11)	0.01 (0.01–44.74)	0.01 (0.01–28.35)
	Sensitivity (95% CI)	0.57 (0.48–0.65)	0.54 (0.45–0.64)	0.60 (0.40–0.77)	0.51 (0.44–0.58)	0.47 (0.42–0.53)	0.55 (0.34–0.74)
	Specificity (95% CI)	0.48 (0.39–0.57)	0.55 (0.47–0.62)	0.39 (0.28–0.51)	0.49 (0.40–0.57)	0.52 (0.44–0.60)	0.41 (0.30–0.54)
Met/Met vs. Val/Met	DOR (95% CI)	1.23 (0.85–1.77)	1.09 (0.68–1.74)	1.48 (0.70–3.17)	1.51 (0.91–2.50)	1.09 (0.55–2.18)	2.36 (0.87–6.40)
	*I^2^* (95% CI)	30.30 (0.01–62.49)	33.5 (0.01–65.62)	0.01 (0.01–74.32)	0.01 (0.01–46.09)	0.01 (0.01–44.88)	0.01 (0.01–0.02)
	Sensitivity (95% CI)	0.26 (0.19–0.35)	0.22 (0.16–0.30)	0.33 (0.19–0.49)	0.31 (0.20–0.45)	0.25 (0.14–0.39)	0.47 (0.27–0.69)
	Specificity (95% CI)	0.77 (0.72–0.82)	0.79 (0.72–0.85)	0.75 (0.65–0.83)	0.77 (0.71–0.81)	0.77 (0.71–0.82)	0.72 (0.63–0.80)
Met/Met vs. Val/Val	DOR (95% CI)	1.41 (0.86–2.31)	1.63 (0.94–2.82)	1.11 (0.42–2.89)	1.59 (0.80–3.17)	1.06 (0.62–1.83)	2.22 (0.69–7.14)
	*I* (95% CI)	54.50 (18.45–74.58)	43.40 (0.01–70.48)	0.01 (0.01–42.66)	4.9 (0.01–38.17)	10.3 (0.01–49.06)	0.01 (0.01–49.76)
	Sensitivity (95% CI)	0.25 (0.14–0.40)	0.21 (0.12–0.35)	0.30 (0.11–0.60)	0.28 (0.15–0.47)	0.19 (0.11–0.30)	0.47 (0.27–0.69)
	Specificity (95% CI)	0.81 (0.71–0.88)	0.86 (0.78–0.91)	0.72 (0.57–0.83)	0.80 (0.70–0.87)	0.82 (0.73–0.88)	0.71 (0.54–0.84)

**Note:** DOR  =  diagnostic odds ratio; CI  =  confidence interval. DOR >1 indicates an increase in violence risk in individuals in the first group in the pairwise comparison with the second. Significance tests only conducted for DORs.

* *p* <0.05.

**Table 4:** Metaregression analyses examining sources of heterogeneity in studies investigating the association between the Met allele of the Val158Met polymorphism and violence.

	Physical violence against others			Homicide only		
	All	Men	Women	All	Men	Women
Sample or study characteristic	*β* (SE)	*β* (SE)	*β* (SE)	*β* (SE)	*β* (SE)	*β* (SE)
**Sex**						
% Men	0.01 (0.01)	–	–	0.01 (0.02)	–	–
Men vs. Women^a^	0.14 (0.10)	–	–	0.07 (0.13)	–	–
**Mean age (years)**						
Continuous	0.01 (0.03)	–	–	–0.02 (0.05)	–	–
**Substance abuse**						
% Alcohol abuse	0.02 (0.01)	–	–	–	–	–
% Drug abuse	0.02 (0.01)	–	–	–	–	–
**Mean time at risk (months)**						
Continuous	0.01 (0.01)	–	–	–0.01 (0.01)	–	–
**Continent of origin**						
Asia vs. Other	0.12 (0.33)	.59 (0.40)	0.64 (0.61)	–0.34 (0.51)	–0.90 (0.77)	–1.42 (1.00)
**Source of outcome**						
Official records only^b^ vs. Other	–0.01 (0.32)	0.05 (0.35)	0.38 (0.59)	–0.42 (0.27)	–0.39 (0.30)	–0.76 (0.99)

**Note:** SE  =  standard error; –  =  analysis not applicable or data not available. Diagnostic odds ratios from the Val/Val vs. Val/Met + Met/Met comparison were used. The moderating role of sample ethnic composition was not investigated due to low levels of within-study ethnic diversity, resulting in overlap with continent of origin. No evidence of significant moderation was found (*p* >0.05 in all cases).

a Study data on men vs. women.

b Institutional and criminal records.

### Systematic search

A systematic search current to November 1, 2011 was conducted using MEDLINE, EMBASE, CINAHL, PsycINFO, ProQuest, and National Criminal Justice Reference Service Abstracts with the following keywords: *Val158Met*, *Val(158)Met, 158 Val/Met*, and *rs4680*. Additional articles were located through reference lists, annotated bibliographies, and discussion with experts. Studies in all languages and those not published in academic journals were considered. Studies were included if their titles, abstracts, or methods sections demonstrated testing of the association between the Met allele of the Val158Met polymorphism of the COMT gene and physical violence against others in schizophrenia.

The initial search identified a total of 2,018 records (Figure 1). The number of records was reduced to 413 when abstracts were scrutinized to see whether they were relevant to individuals with schizophrenia. When editorials, reviews, and reports were excluded that did not use physical violence against others as an outcome, 17 independent studies remained.

Inclusion in the meta-analysis required that the number of participants with Val/Val, Val/Met, and Met/Met genotypes be available, as well as the number of individuals within each genotype that engaged in physical violence against others. Such tabular data were either available in the manuscript or obtained directly from authors for 15 (88.2%) of the 17 eligible studies, 14 (93.3%) of which also contributed specific information on homicide as an outcome, and 13 (86.7%) of which contributed outcome data separately for men and women. As this data concerned only those participants diagnosed with schizophrenia and the published data often included persons with other schizophrenia-spectrum disorders, the sample sizes, number of participants with each genotype, and base rates of violence used in the present review often differ from those published in the original manuscripts. The two studies for which tabular data were unobtainable [10], [13] were excluded from analyses. Thus, a total of 15 studies were included.

### Data extraction

JS extracted 39 demographic and descriptive characteristics from each of the included studies. As a measure of quality control, five (33.3%) of the studies were randomly selected and coded by a second investigator (PC). A high level of inter-rater agreement was established (κ = 0.93), and disagreements were settled by consensus [14].

### Meta-analysis

Following current Cochrane Collaboration guidance [15], bivariate analyses of study sensitivities (the proportion of violent individuals classified as “high risk”) and specificities (the proportion of non-violent individuals classified as “low risk”) were conducted, accounting for correlation between these study values [16]. This methodology offered an alternative parameterization of the hierarchical summary receiver operating characteristic (HSROC) model [17], and allowed for the identification of a summary operating point from which pooled diagnostic odds ratios (DOR; the ratio of the odds of a true positive relative to the odds of a false positive) were calculated along with pooled sensitivities and specificities. The DOR is not only an appropriate effect size when a specific diagnostic marker or test is used to detect the presence of an adverse state (e.g., using a mammogram to detect breast cancer), but also in general contexts such as the present meta-analysis where the association between dichotomous independent and dependent variables is tested (see *Genotypic comparisons*) [18]. When the latter conditions are met, the DOR is equivalent to the odds ratio obtained using logistic regression [18]. The percentage of variation across study DORs not due to chance alone was estimated using the *I^2^* index [19]. As the HSROC model estimates summary DORs using a different parameterization than the traditional Mantel-Haenzel and DerSimonian-Laird models, summary ROC curves were constructed to graphically display individual study and pooled DORs rather than forest plots. Diagnostic methodology such as that employed in the present review has been used in recent meta-analyses for the investigation of the association between common SNPs and other complex behaviors such as suicide [20], substance abuse [21], and antisocial behavior amongst maltreated children [22].

### Genotypic comparisons

The hypothesis that the presence of a single Met allele would result in elevated violence risk was meta-analytically investigated. Individuals with Val/Met or Met/Met genotypes were combined and compared with individuals with the Val/Val genotype. This approach, adopted *a priori,* was based on an analogous meta-analysis of COMT polymorphism and suicide [20], and two publications included in the current review [23], [24].

Two alternative genotypic comparisons were planned *a priori* to explore the effect of the Met allele in subsets of participants: First, heterozygous Val/Met individuals were compared to homozygous Met/Met individuals to examine whether a dose-response effect existed. Second, individuals with the Val/Val genotype were compared to individuals with the Met/Met genotype to examine whether the Met allele increased violence risk, in the absence of the potential effects of genetic heterozygosity [25], [26].

### Sources of between-study heterogeneity

Random effects metaregression analyses were conducted to investigate whether sample or study characteristics were associated with variation in DORs [27]. We explored the moderating influence of the following: sex, age, substance abuse, time at risk, study continent of origin, and source of outcome information. Sex was analyzed as both a continuous (percentage of sample who were men) and categorical (study data for men vs. women) variable. Substance abuse (percentage of sample who had lifetime diagnoses of alcohol or drug abuse), sample age (mean in years), and time at risk (mean in months) were investigated continuously. Continent of origin (Asia vs. other) and source of outcome information (official records only [institutional and/or criminal] vs. official records and self- or collateral-report) were explored categorically. It was decided *a priori* to conduct metaregression analyses regardless of heterogeneity levels, as current expert opinion dictates that sources of heterogeneity should be investigated regardless of between-study variability levels [28].

### Assessment of publication bias

Publication bias is assessed routinely using statistical analogues of funnel plots [29]. In line with current Cochrane Collaboration guidance [15], we used a recently developed modified linear regression test, based on the efficient score and its variance [30], to assess evidence of publication bias. This novel test was selected, as commonly used tests to detect funnel plot asymmetry have been shown to result in elevated false positive rates when applied to binary outcome data. We did not construct a funnel plot, as such visual tests produce high false positive rates when DORs are used as outcome measures [15].

A significance level of α = 0.05 was adopted for all analyses.

## Results

### Descriptive characteristics

Information was collected on 2,370 individuals with schizophrenia from 15 retrospective investigations of the association between the Val158Met polymorphism of the COMT gene and physical violence against others. One article [31] was in Chinese and was therefore translated by a research assistant working independently of the authors. The average sample size was 158 (SD  = 130) participants, with a trend towards predominantly male samples. An average of 76 (SD  = 69) participants per study had the Val/Val genotype, 62 (SD  = 57) the Val/Met genotype, and 20 (SD  = 15) the Met/Met genotype, with Hardy-Weinberg equilibrium achieved in all investigations. Participants were recruited from inpatient hospitals in 10 (66.7%) studies, from the community in 1 (6.7%) study, and from both inpatient and community settings in 4 (26.7%) studies. The average duration of psychotic symptoms at the time of genotyping was 15.7 years (SD = 7.7).

Over a mean time at risk of 136.7 (SD  = 184.4) months, 951 (40.1%) of the 2 370 participants were violent, with 460 (20.7%) committing homicide. Six (40.0%) investigations used community offending as their outcome, whereas one (6.7%) used institutional offending, and eight (53.3%) used offending in either setting. Four (26.7%) studies relied upon institutional records to ascertain whether participants had been violent, 1 (6.7%) relied upon criminal records, and the remaining 10 (66.7%) used a combination of institutional and criminal records along with self- and collateral-report. Whether violent individuals were on antipsychotic medication at the time of their offense was only ascertainable from three studies [31]–[33] with an average of 28.5% (SD  = 2.1%) of participants meeting this criterion. Additional demographic and study design characteristics are provided in Table 1, and outcome characteristics are reported in Table 2.

### Genotypic meta-analyses

Evidence of a significant association between the presence of a Met allele and violence was found such that men's violence risk increased by approximately 50% for those with at least one Met allele compared with homozygous Val individuals (DOR  =  1.45; 95% CI  = 1.05–2.00; *z*  =  2.37, *p*  = 0.02; Figure 2). However, the association was not significant when violence was restricted to homicide (DOR  = 0.96; 95% CI  = 0.71–1.30; *z*  = 0.24, *p*  = 0.81), suggesting the Met allele may be associated with less serious physical violence. No significant association between the presence of a Met allele and violence was found for women or when men and women were combined (Table 3). Further, no significant dose-response relationships were found when heterozygous individuals were compared with homozygous Met individuals.

When homozygous individuals were compared, high rates of specificity (range  = 0.72–0.86) were found. While the association with violence in the pairwise comparison between the Met homozygous and Val homozygous groups did not reach our threshold of statistical significance, a clear trend was identified in the anticipated direction (DOR  = 1.63; 95% CI  = 0.94–2.82; *z*  = 1.55, *p*  = 0.06).

### Investigation of heterogeneity

Low to moderate levels of between-study heterogeneity were found, with the upper-limits of all *I^2^* estimate confidence intervals below 75% [34]. The source of this heterogeneity was investigated using metaregression (Table 4). Using outcome data for all participants, a marginally significant trend was found such that in samples with higher rates of lifetime drug (non-alcohol) abuse, individuals with the Met allele were at higher risk of violence (*β* = 0.02, *p* = 0.09). No statistically significant moderators were identified using data from the alternative genotypic comparisons.

### Investigation of publication bias

No significant evidence of publication bias was found in any of the genotypic comparison datasets (test of funnel plot asymmetry *p*>0.05 in all cases).

## Discussion

We conducted a meta-analysis involving 2,370 individuals with schizophrenia. We were able to use 15 of the 17 (88%) eligible retrospective investigations that assessed the association between the Val158Met polymorphism of the COMT gene and physical violence against others.

Our principal finding is that the presence of one or more Met alleles in the COMT genotype elevates interpersonal violence risk in male schizophrenia patients. The Met allele codes for the low activity form of the enzyme, and thus this finding is consistent with the report of elevated aggressivity in male COMT knockout mice [8]. The principal finding is also consistent with the preponderance of other evidence supporting the roles of dopamine and noradrenaline in the biology of violence.

The role of many other biological factors in violence in schizophrenia is well documented [35]. Furthermore, the effects of the rearing environment, various sociodemographic factors, history of conduct disorder, stress, current psychotic symptoms, comorbid substance use, comorbid personality disorders, and factors related to treatment constitute a network of interacting pathways leading to violence in schizophrenia [36].

Some of these factors may be related to the COMT polymorphism. Met/Met homozygotes have been found to be at significantly increased risk of behavioral and emotional disturbances such as impulsiveness and conduct problems at the ages of 7 and 11 years, relative to either heterozygous or homozygous Val carriers of the Val158Met polymorphism, but only when they were exposed to maternal stress in utero [37]. Conduct disorder is a known precursor of violence in schizophrenia [36], [38], [39]. Patients with “non-affective psychotic disorder” who were Met/Met homozygotes have been shown to exhibit significantly increased psychotic and affective reactivity to stress in comparison to the Val/Met and Val/Val genotypes. In contrast, healthy controls have not shown this effect of the COMT polymorphism [40]. Parental violent crime is associated with violent crime in offspring with schizophrenia, which suggests a role of familial (genetic or early environmental) factors in this transmission [41]. The molecular basis of this effect has not been explored.

Impulsivity and violence are related to suicide risk in male schizophrenia patients [42]. Both suicidal [43] and violent [44] behaviors in schizophrenia respond to clozapine. Suicidal and violent behaviors share certain neurobiological features [2]. It is therefore of interest that the Met allele in the COMT genotype was associated with the history of violent suicide attempts in schizophrenia patients [45] and with suicide attempts in alcoholics [46]. These findings are consistent with our results.

We were able to obtain limited information regarding patients' antipsychotic treatment at the time of their violent incidents. Less than one-third were reported to be receiving such treatment at the time of their violence, and this may be an over-estimate, given the fact that at least 40% of schizophrenia patients are non-adherent to treatment [47]. Our finding is consistent with the general agreement that schizophrenia patients who are off medication are at elevated violence risk [2], [48]–[50].

There was a marginal trend for higher violence risk in individuals with the Met allele in samples with higher rates of lifetime drug (non-alcohol) abuse. Substance abuse elevates the risk of violence in schizophrenia [51], and it is possible that this effect is partially moderated by the COMT polymorphism. However, the information on substance abuse in the reports we analyzed was incomplete, and our finding was not statistically significant. This should be followed up in future prospective studies.

No significant association between the presence of the Met allele and violence was found when outcome was restricted to homicide. With a subset of 460 homicide cases in our sample, if the Met allele were strongly associated with homicide in the population, one would expect that such an effect would have been detectable in a subset of this size. Although we cannot exclude the possibility of a Type II error, it is possible that the genetic underpinning of homicide is not identical with that of less severe forms of violence. Specifically, a functional single nucleotide polymorphism (Ala72Ser) in the COMT gene has been shown to differentiate schizophrenia patients who commit homicide [52]. Thus, the lack of an association between the Met allele and homicide that we observed may reflect a biological distinctiveness of this specific criminal behavior in schizophrenia.

The present study has several limitations in addition to the lack of information on comorbid substance abuse. First, while no evidence of a significant association between the presence of a Met allele and violence against others was found in women, this is based on eight (53.3%) predominantly male samples. Future studies may wish to further investigate the effect of the Met allele in women only. A second limitation was that, as detailed previously, two studies that met inclusion criteria had to be excluded, as the reports commingled physical violence against others with other antisocial behaviors. One of these excluded studies, using 37 patients, reported an association between the Met allele and generally “aggressive and antisocial behavior” [10]. The other study, using 70 subjects, reported no association between the COMT polymorphism and violence, inclusive of verbal aggression and physical violence against objects [13]. Thus, the results of these two excluded studies came to different conclusions regarding the association between the COMT polymorphism and violence. However, both studies were relatively small, leading us to believe that their exclusion was not likely to have appreciably affected our results. A third limitation was that population stratification due to unmeasured sample-level characteristics may have influenced effect sizes. Although metaregression was used to explore the potentially moderating role of geographic location, sex, and age with no significant findings, future studies may wish to investigate whether additional characteristics such as immigrant status and parental country of origin influence the association between the presence of the Met allele and violence in schizophrenia.

The principal strength of our meta-analysis is its narrow focus. We have limited the diagnosis to schizophrenia, and the behavior of interest to physical violence against others. Self-harm, violence against objects, verbal aggression, as well as threats and other antisocial behaviors were excluded. These behaviors have often been commingled with physical violence in previous studies. One of the reasons for variability in the findings of past studies of this type may have been between-study heterogeneity in diagnostic criteria and the operationalization of violence.

Our findings may have several implications for treatment. Clozapine is currently the most effective treatment of violent behavior in schizophrenia patients [44]. Nevertheless, many patients fail to respond, discontinue the treatment, or experience prohibitive side effects [53]. Beta-adrenergic blocking agents could be a suitable alternative or adjunctive treatment in such cases. Older studies reported encouraging results in violent schizophrenia patients [5], [6], but the treatment has been virtually abandoned. This was partly due to the blocking agents' cardiovascular side effects, necessitating a slow dose escalation rate, and partly to the pressure to use newly introduced second generation antipsychotics. However, if violent behavior is due, in part, to enhanced noradrenergic activity in some patients with schizophrenia, then beta-adrenergic blocking agents could be effective in these particular patients. Theoretically, male patients possessing at least one Met allele in their COMT genotype should be the suitable candidates for such treatment. Future randomized controlled trials of beta-adrenergic blocking agents using COMT genotype as a predictor variable could test this hypothesis.

Although we have narrowed our violent phenotype as much as possible given the retrospective nature of our study, future prospective research is warranted to examine the association between the Met allele and violence in men with psychosis. Violence against others in schizophrenia has several subtypes that differ in their pathogenesis, treatment, and prognosis [39], [44], [54]. The role of the COMT genotype likely varies across such subtypes, and this variation should be investigated.

We conclude that male schizophrenia patients who carry the low activity Met allele in the COMT gene are at a modestly elevated risk of violence. This finding has potential implications for the pharmacogenetics of violent behavior in schizophrenia.
